# Severe maternal morbidity in Zanzibar’s referral hospital: Measuring the impact of in-hospital care

**DOI:** 10.1371/journal.pone.0181470

**Published:** 2017-08-23

**Authors:** Tanneke Herklots, Lieke van Acht, Tarek Meguid, Arie Franx, Benoit Jacod

**Affiliations:** 1 Division of Woman and Baby, University Medical Centre Utrecht, Utrecht, The Netherlands; 2 Department of Obstetrics and Gynaecology, Mnazi Mmoja Hospital, Stone Town, Zanzibar, United Republic of Tanzania; 3 School of Health & Medical Sciences, State University of Zanzibar (SUZA), Zanzibar, United Republic of Tanzania; 4 Department Obstetrics & Gynaecology, Radboud University Medical Centre, Nijmegen, The Netherlands; Centre Hospitalier Universitaire Vaudois, FRANCE

## Abstract

**Objective:**

to analyse the impact of in-hospital care on severe maternal morbidity using WHO’s near-miss approach in the low-resource, high mortality setting of Zanzibar’s referral hospital.

**Setting:**

Mnazi Mmoja Hospital, a tertiary care facility, in Zanzibar, Tanzania.

**Methods:**

We identified all cases of morbidity and mortality in women admitted within 42 days after the end of pregnancy at Mnazi Mmoja Hospital in the period from April to October 2016. The severity of complications was classified using WHO’s near-miss approach definitions: potentially life-threatening condition (PLTC), maternal near-miss (MNM) or maternal death (MD). Quality of in-hospital care was assessed using the mortality index (MI) defined as ratio between mortality and severe maternal outcome (SMO) where SMO = MD + MNM, cause-specific case facility rates and comparison with predicted mortality based on the Maternal Severity Index model.

**Main outcomes:**

5551 women were included. 569 (10.3%) had a potentially life-threatening condition and 65 (1.2%) a severe maternal outcome (SMO): 37 maternal near-miss cases and 28 maternal deaths. The mortality index was high at 0.43 and similar for women who developed a SMO within 12 hours of admission and women who developed a SMO after 12 hours. A standardized mortality ratio of 6.03 was found; six times higher than that expected in moderate maternal mortality settings given the same severity of cases. Obstetric haemorrhage was found to be the main cause of SMO. Ruptured uterus and admission to ICU had the highest case-fatality rates. Maternal death cases seemed to have received essential interventions less often.

**Conclusions:**

WHO’s near-miss approach can be used in this setting. The high mortality index observed shows that in-hospital care is not preventing progression of disease adequately once a severe complication occurs. Almost one in two women experiencing life-threatening complications will die. This is six times higher than in moderate mortality settings.

## Introduction

Maternal mortality is largely preventable. It represents the most visible result of shortcomings of maternal health care systems in many low and middle income countries [1]. It has been targeted by the Millennium Development Goals until 2015 and by the Sustainable Development Goals since. These efforts have contributed to a significant decrease in the last years although this decrease has not been uniform across regions and subsets of populations at risk [2].

The improvement of maternal healthcare is partly due to the encouragement of attendance of deliveries by skilled-birth attendants and of deliveries in health facilities [3]. The growing numbers of deliveries now taking place in health facilities imply that the quality of care received at that level has an increasing impact on eventual overall health gains. Maternal death reviews are one way of identifying possible deficiencies in maternal care [4]. Their low number at facility level, even in high mortality settings, may however hamper the generalization of their findings. A complementary approach is to consider severe maternal morbidity. Its definition is, however, not unequivocal [5] and might not always represent the same pathways as that leading to maternal death [6].

The World Health Organization (WHO) has proposed a framework for the analysis of severe maternal morbidity [7]. It defines categories of morbidity with increasing severity: potentially life-threatening conditions (PLTC), life-threatening conditions or maternal near-miss (MNM) and maternal death (MD) [6]. Although some doubts about its applicability have been raised at facility levels [8,9] there is now a large amount of data from a variety of settings suggesting that it is a workable approach [10–18]. It enables to quantify quality of care by comparing the observed frequency of adverse outcomes to predictions based on an assessment of severity of the underlying conditions [12,19]. On a qualitative level, it provides a subset of cases of maternal near-miss that can be audited in addition to maternal death cases.

This article presents an evaluation of WHO’s maternal near-miss approach in a single, tertiary care facility in a low-income, high-maternal mortality context. Mnazi Mmoja Hospital (MMH) is the referral hospital in Zanzibar, on Unguja island, with around 13,000 deliveries per year. The availability of essential interventions and an intensive care unit is mostly adequate despite episodic shortages. Thanks to the island size and good overall infrastructure, delays in referral and consultation are less substantial than in other low-income settings. As a consequence, the impact of in-hospital care on overall maternal outcomes is significant. The current article illustrates how this impact can be measured using WHO’s near-miss approach.

## Methods

We performed a cross-sectional analysis of maternal and perinatal outcomes of all women admitted at Mnazi Mmoja Hospital while pregnant or within 42 days after delivery or end of pregnancy from April to October 2016. The study was approved by Zanzibar’s Medical Ethical Research Committee (ZAMREC/0001/AUGUST/005). Informed consent was waived because the study concerned only an analysis of clinical files with aggregated, anonymous outcomes.

Cases of severe maternal morbidity were identified daily by collecting files after discharge of the obstetrics and gynaecology department, intensive care unit (ICU) and emergency service of every woman, pregnant or within 42 days after the end of pregnancy. Data collection was performed by two junior investigators (TH and LA) who were not involved in the delivery of care. Demographics, maternal and perinatal outcomes were collected from all files. Age and parity categories were chosen based on considered clinical relevance, with age cut-offs <20 years, 20–35 years and >35 years, and parity cut-offs at nulliparous, parity of 1–4 and parity > 4, being grand multiparous women. The data has further been categorized in complications occurring before 22 weeks of gestation and later. The cut-off of 22 weeks has been chosen to distinguish between the type of complications occurring in the first half of the pregnancy, e.g. complications related to abortion or ectopic pregnancies, and the complications of the second half of pregnancy, such as pre-eclampsia. Although we acknowledge that other distinctions could be made, for instance in terms of viability at gestational age of 28 weeks.

Cases with potentially life-threatening conditions were analysed further using WHO’s near-miss approach. When clarification was needed, clinicians involved in cases of MNM were interviewed. In case of disagreement or doubt, the judgement of a third assessor (TM or BJ, both consultants in Obstetrics and Gynaecology) was decisive (n = 24). All cases of maternal death were routinely audited within 72 hours of the event by the multi-disciplinary local maternal death review team.

Data analysis was performed by a junior (TH) and senior investigator (BJ) following WHO’s near-miss approach [7] with all cases classified either as a non-complicated pregnancy, a pregnancy complicated by a potentially life-threatening condition (PLTC), a maternal near-miss (MNM) or a maternal death (MD). According to this approach, PLTC cases were considered as such when the woman had severe postpartum haemorrhage, severe pre-eclampsia, eclampsia, sepsis or severe systemic infection, uterus rupture or when one of the following interventions was performed: use of blood products, laparotomy, admission to ICU. Maternal near-misses were considered as such when there was at least one marker for organ dysfunction (see S1 Table). Some of these markers were not applicable to the setting of MMH, which is indicated in the table. Furthermore, in a context of relative scarcity of blood products, we adapted the clinical criteria for coagulation dysfunction from 5 or more units of packed red cells transfused to a total of 5 or more units of blood products transfused or ordered but not entirely given due to shortage, therefore also including free frozen plasma and thrombocyte concentrate.

Due to the descriptive character of this study, the sample size was not powered for. Based on the estimated MMRs of previous years, and the planned 6-months duration of the study, we expected roughly 6,000 patients, including 30 maternal deaths, to be included. Descriptive statistics were used to calculate the frequencies of baseline characteristics according to maternal outcome: either no severe maternal morbidity, i.e. no maternal near-miss and no maternal death (non-SMO), or with a maternal near-miss or maternal death (SMO). Due to limited documentation, only women’s age, parity, gestational age on admission, mode of delivery and vital status of the infant at birth could be evaluated. The non-SMO- and SMO-group were compared and p-values were calculated by using Pearson’s chi-square test or, in case of small sample size, the Fisher exact test, with p-values below 0.05 considered to be statistically significant.

The outcome measures are described in Table 1. Overall morbidity was evaluated by the calculation of ratios for the different groups within the spectrum of morbidity: PLTC, SMO, MNM and MD following WHO’s definitions. The quality of care was evaluated by considering the mortality index (MI: MD divided by SMO), cause-specific case fatality rates (CFR) and the standardised mortality ratio (SMR). The SMR was derived from the maternal severity index (MSI)-model [19]. This model calculates an expected maternal mortality based on the number of severity markers observed in the population. It has been developed in a moderate maternal mortality setting. The standardized mortality ratio is the ratio between observed and predicted maternal mortality risk. Additionally, the mortality index was calculated separately for women developing SMO within 12 hours of referral and later to assess the impact of referral and pre-hospital delay [5].

**Table 1:** Outcome measures.

Measure	Description
**Ratio of potentially life-threatening conditions (PLTC)**	Number of PLTC cases per 1000 live births
**Severe maternal outcome (SMO) ratio**	Number of cases of maternal near-miss and maternal death per 1000 live births
**Maternal near-miss (MNM) incidence ratio**	Number of cases of maternal near-miss per 1000 live births
**Maternal near-miss mortality ratio**	Ratio between number of maternal near-miss cases and number of maternal deaths
**Maternal mortality ratio (MMR)**	Number of maternal death cases per 100,000 live births
**Observed mortality index (MI)**	Number of maternal death cases divided by the total number of cases with a severe maternal outcome
**Maternal severity score**	Total number of severity markers in all SMO cases divided by the total number of women with a SMO, resulting in the average number of severity markers per SMO case
**Mean maternal severity index (MSI)**	The case-specific maternal severity index as calculated per SMO case with the MSI calculator [8], after which the mean of all 65 MSI’s was calculated
**Standardised mortality ratio (SMR)**	As calculated with the SMR calculator [8] using this study’s specific mean MSI, indicating a woman’s risk of dying

Data collection took place through KoBoToolbox after which it was anonymised and saved in a database using Microsoft Office Excel (2007). OpenEpi was used for statistical analysis.

## Results

**Fig 1:**
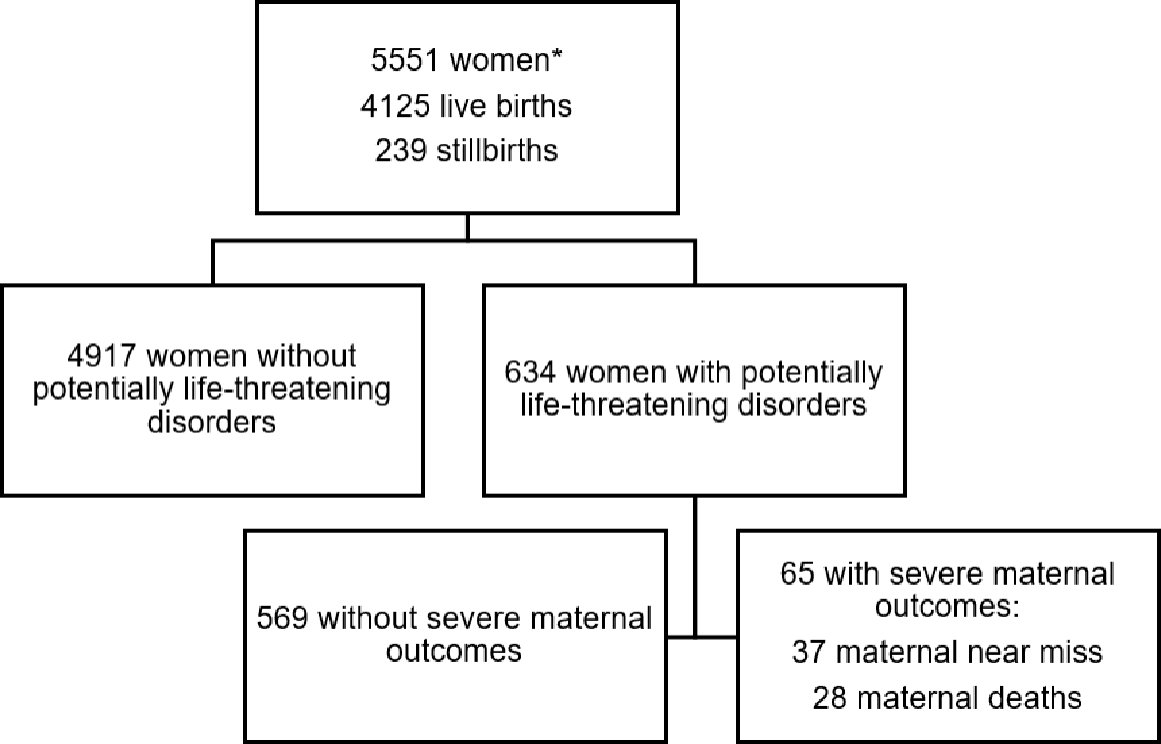
Study profile. *) Sum of live births and stillbirths does not equal the total number of women because some of the included women had an abortion, did not deliver or data was missing from the patient file.

During the six-month data collection period, 5551 women admitted at MMH–pregnant or within 42 days after end of pregnancy–were included. There were 4527 deliveries and 4125 recorded live births. There were 239 stillbirths, giving a stillbirth rate of 52.8 per 1000 births. Of all women, 634 had a (potentially) life-threatening condition, of which 65 women had a severe maternal outcome, with 37 maternal near-miss cases and 28 maternal deaths, see Fig 1. Table 2 contains general characteristics of the study population according to maternal outcome. The percentage of available data is included in the table since missing data was an evident phenomenon, ranging from 1% for age to 7.1% for type of delivery. There is a trend towards a larger proportion of older women in the SMO group although this does not reach significance. Compared to non-SMO, women with SMO were significantly more likely to be multiparous, 80% SMO vs. 56.8% non-SMO, to have been admitted post-partum, 14.1% vs. 1.7%, to have delivered by caesarean, 63% vs. 13.1%, and to have delivered a stillborn child, 38.3% vs. 5.1%.

**Table 2:** Baseline characteristics, labour and perinatal outcomes of women according to maternal outcome.

	All women	Women without SMO	Women with SMO	p-value
	N (%)	N (%)	N (%)	
**Total**	5551 (100)	5486 (98.8)	65 (1.2)	
**Age**				p = 0.06
Data available	5494 (99.0)	5429 (99.0)	65 (100.0)	
<20 years	510 (9.3)	502 (9.2)	8 (12.3)	
20–35 years	4276 (77.8)	4233 (78.0)	43 (66.2)	
>35 years	708 (12.9)	694 (12.8)	14 (21.5)	
**Parity**				p<0.001
Data available	5367 (96.7)	5302 (96.6)	65 (100.0)	
0	2301 (42.9)	2288 (43.2)	13 (20.0)	
1–4	1495 (27.9)	1456 (27.5)	39 (60.0)	
>4	1571 (29.2)	1558 (29.3)	13 (20.0)	
**Gestational age on admission**				p<0.001
Data available	5464 (98.4)	5400 (98.4)	64 (98.5)	
<22 weeks	460 (8.4)	453 (8.4)	7 (10.9)	
>22 weeks	4900 (89.7)	4852 (89.9)	48 (75.0)	
Post-partum	104 (1.9)	95 (1.7)	9 (14.1)	
**Type of delivery**				
Data available	5160 (93.0)	5096 (92.9)	63 (97.0)	
GA <22 weeks	383 (7.4)	378 (7.3)	5 (7.9)	
• Complete abortion or curettage	360 (94.0)^a^	355 (93.9)^a^	5 (100)^a^	
• Laparotomy for ectopic pregnancy	23 (6.0)^a^	23 (6.1)^a^	0 (-)	
GA >22 weeks	4527 (87.8)	4481 (87.9)	54 (70.8)	
• Spontaneous vaginal delivery	3879 (85.7)^b^	3865 (86.3)^b^	19 (35.2)^b^	p<0.001^c^
• Caesarean delivery	620 (13.7)^b^	589 (13.1)^b^	34 (63.0)^b^	
• Instrumental vaginal delivery	28 (0.6)^b^	27 (0.6)^b^	1 (1.8)^b^	
Discharged/died still pregnant	250 (4.8)	246 (4.8)	4 (6.3)	
**Vital status of infant at birth**^**d**^				p<0.001
Data available	4364 (96.4)	4317	47	
Live births	4125 (94.5)	4096 (94.9)	29 (61.7)	
Stillbirth	239 (5.5)	221 (5.1)	18 (38.3)	

a) calculated as a percentage of total number of deliveries with gestational age below 22 weeks

b) calculated as a percentage of total number of deliveries with gestational age of 22 weeks or higher

c) vaginal delivery (including instrumental delivery) versus caesarean delivery

d) only for pregnancies above 22 weeks of gestation (n = 4527)

Maternal outcomes are shown in Table 3. The population is characterized by a high severity with a morbidity rate, combined of the PLTC- and SMO rates, of 154 per 1000 live births. Over 10% of these women developed SMO (9 MNM and 7 MD per 1000 live births). The institutional maternal mortality ratio (MMR) was 647 per 100.000 livebirths. For every 10 cases of maternal death there were 13 maternal near-miss cases corresponding to a mortality index of 0.43. About two thirds of women developed SMO within 12 hours of admission. This group had a mortality index of 0.45, similar to that of women developing SMO later than 12 hours after admission (0.39).

**Table 3:** Severe maternal outcomes, morbidity and mortality indicators and facility-related indicators.

Maternal outcomes	N (% of total)
Women with potentially life-threatening complications^a^	569 (10.3)
Severe maternal outcome cases	65 (1.2)
Maternal near-miss cases	37 (0.7)
Maternal death cases	28 (0.5)
**Overall severe morbidity, near-miss and mortality indicators**	
Ratio of potentially life-threatening conditions (per 1000 live births)	138
Severe maternal outcome ratio (per 1000 live births)	16
Maternal near-miss incidence ratio (per 1000 live births)	9
Maternal near-miss mortality ratio	1.3:1
Maternal mortality ratio (per 100,000 live births)	647
Observed mortality index (MD/SMO)	0.43
Maternal severity score (average no of severity markers in all SMO cases)	2.66
Mean maternal severity index (%)^b^	7.32
Standardised mortality ratio (observed MI/predicted MI)^c^	6.03 (95% CI 2.48–14.68)
**Hospital access indicators**	
SMO cases within 12h of hospital stay (% of all SMO)	42 (65%)
Percentage of SMO cases within 12h of hospital stay among admissions from other health facilities (n = 230)	11/230 = 4.9%
Mortality index for SMO cases within 12h of hospital stay	19/42 = 0.45
**In-hospital care**	
SMO cases developed after 12h of hospital stay	23
In-hospital mortality index	9/23 = 0.39

a) excludes SMO.

b) calculated per SMO case with MSI calculator [8], after which the mean of all 65 MSI’s was calculated.

c) as calculated with SMR calculator [19] using this study’s specific mean MSI.

The severity score of women developing SMO is 2.66, meaning that on average every woman with SMO showed 2.66 organ dysfunction markers. The MSI-model was used to estimate the number of women that would die if those complications had occurred in a health care environment found in most moderate mortality settings. The SMR was subsequently calculated by dividing the number of observed deaths by the number of expected deaths. For Mnazi Mmoja Hospital this ratio is 6.03, meaning that 6 times more maternal deaths occurred than would have been expected assuming the level of care found in moderate MMR settings.

In Table 4, the distribution of potentially life-threatening disorders is presented per maternal outcome group. Obstetric haemorrhage and hypertensive disorders were the most frequent complications in all groups. Their relative weight, however, differed significantly per group. Obstetric haemorrhage was found to occur in 46.4% of maternal deaths and 29.7% of MNM while it only represented 15.1% in the PLTC group. By contrast, severe pre-eclampsia was present in 25.8% of PLTC but only in 13.5% of MNM and 21.4% of maternal deaths. Finally, ruptured uterus was shown to have the highest case fatality rate of 27.3% while severe complications of abortion had the lowest at 2.4%. Critical interventions such as laparotomy (excluding caesarean delivery) and ICU admission occurred as expected much more often in the SMO group than in the PLTC group. Admission to ICU had the highest case fatality rate of all at 33.3%.

**Table 4:** Frequency of potentially life-threatening disorders.

	PLTC	MNM	MD	CFR^a^
	N (%)	N (%)	N (%)	(%)
**Total**	569 (100)	37 (100)	28 (100)	-
**Severe complications**				
Severe pre-eclampsia	147 (25.8)	5 (13.5)	6 (21.4)	3.8
Eclampsia	49 (8.6)	4 (10.8)	2 (7.1)	3.6
Severe post-partum haemorrhage	86 (15.1)	11 (29.7)	13 (46.4)	11.8
Ruptured uterus	5 (0.9)	3 (8.1)	3 (10.7)	27.3
Sepsis or severe systemic infection	29 (5.1)	4 (10.8)	3 (10.7)	8.3
Severe complication of abortive, ectopic or molar pregnancy	78 (13.7)	3 (8.1)	2 (7.1)	2.4
Other/none	-	1 (2.7)^b^	-	-
**Critical interventions**				
Blood transfusion	331 (58.2)	30 (81.1)	14 (50.0)	3.7
Laparotomy	26 (4.6)	13 (35.1)	11 (39.3)	22.0
Admission to ICU	9 (1.6)	21 (56.8)	15 (53.6)	33.3
Contributing factors				
Anaemia	295 (51.8)	26 (70.3)	14 (50.0)	
Previous caesarean section	48 (8.4)	5 (13.5)	4 (14.3)	
HIV	4 (0.7)	-	-	

a) The case fatality rate, CFR, is the calculated percentage of maternal deaths of the total number of women with the severe complication or critical intervention (MD/(PLTC+MNM+MD)).

b) 1 patient with a severe asthma attack post-partum.

Table 5 shows the coverage of key interventions according to maternal outcome. In the PLTC group coverage is generally high, above 85% of the target population. The degree of coverage seems to decrease when severity increases. Maternal death cases seem to have received essential interventions less often. Substantial underreporting is, however, likely to play a role here while the small absolute number of cases makes any firm conclusions difficult.

**Table 5:** Coverage of key interventions*.

	All delivered women	PLTC	SMO	MD
	%	%	%	%
Prophylactic oxytocin. *Target group*: *all delivered women with gestational age ≥ 22wk (n = 4527)*	89.5	85.8	89.2	90.9
Therapeutic oxytocin. *Target group*: *severe pph (n = 110)*		86.0	45.8	38.5
Prophylactic magnesium sulphate. *Target group*: *severe pre-eclampsia (n = 158)*		85.7	81.8	66.7
Therapeutic magnesium sulphate. *Target group*: *eclampsia (n = 54)*		91.8	83.3	50.0
Parenteral antibiotics. *Target group*: *sepsis (n = 37)*		96.7	71.4	66.7

*) percentage calculated as number of women that received the intervention divided by number of women in the specific subgroup (PLTC/SMO/MD) of the target group x 100.

## Discussion

This study aimed to evaluate the applicability of WHO’s near-miss approach in MMH and its assessment of the quality of in-hospital care. With minimal adjustments, it has found to be applicable to this setting. It shows that in-hospital care is of low quality with an observed maternal mortality that is six times higher than expected in moderate mortality settings with similar severity. Admission to the ICU, post-partum haemorrhage and uterus rupture have the highest case-fatality rates.

The WHO is advocating a uniform approach in defining and analysing maternal near-miss to foster comparison. This approach has been developed and tested in middle income countries and has since then shown to be applicable over a wide range of conditions in both large (multi)national trials [10,12,16,20] and at single institutions [9,11,14,15,17,21,22,23]. A main point of discussion is whether, in resource poor environments, it does not lead to underreporting because of restrictions in applicability of some, most commonly laboratory, criteria [8,9,22]. Even when nominally available, those criteria tend to be used significantly less often in high-mortality countries than in moderate to low mortality settings [11,12]. Another issue is the limited availability of blood transfusion which might lead to missing near-miss cases because the criteria of 5 or more PRC transfused can’t be reached [9]. Both issues apply to the current study where some laboratory criteria were not available and a modified definition of massive haemorrhage was used. The impact thereof on the measured incidence of maternal near-miss is difficult to ascertain. However, the rate found in Zanzibar compares well with a large study performed under similar conditions (large, tertiary care facility) in another East African country [14] and lies between the average MNM rate found in high mortality (6.2 per 1000 live births) and very-high mortality (13.1 per 1000 live births) countries in the Multicountry Survey study [12]. However, a similar study in a high mortality country has shown a much higher MNM rate [7]. An overview of studies published with comparable MMR and incidence of potentially life-threatening conditions is shown in the appendix (S2 Table).

Mnazi Mmoja Hospital has a very high mortality rate of 647 per 100,000 live births. Women die in Zanzibar, as in other low-income, high mortality countries, mostly through haemorrhage and hypertensive disorders. Together, these two account for two thirds of all cases of maternal death. In terms of risk assessment, when complications develop, women experiencing uterus rupture are most likely to die from it, followed by severe post-partum haemorrhage. Hypertensive disorders, while frequent, have a lower case-fatality ratio. These findings imply that adequate monitoring of labour progress and a heightened attention to the post-partum period are interventions very likely to have a clinical impact in the hospital setting. Adequate antenatal controls might on the other hand reduce the number of complications due to hypertensive disorders. The coverage of essential interventions seems reasonable but, as underline by Souza et al. [12], might not be an adequate measure of quality of care. In the context of overcrowded, understaffed delivery rooms, acute complications require the type of care–quick, adequate, comprehensive–least likely to be received. The near-miss approach complements findings of maternal death reviews by covering in the whole spectrum of morbidity. It shows that this cannot be explained only by the case-mix as other settings with similar incidence of potentially life-threatening conditions achieve much lower maternal mortality [12,16]. The maternal mortality index is high at 0.43 implying that once life-threatening conditions develop, in-hospital clinical care is unable to prevent further deterioration. This ratio might be slightly inflated due to expected underreporting of maternal near-miss cases, although we found it to be comparable to mortality ratios found in comparable settings. Oladapo et al. [10] report a MI of 0.41 in a multicentre study performed in tertiary care facilities in Nigeria while Tunçalp et al. [11] report a MI of 0.28 in a single centre tertiary care facility in Ghana. Note that in the latter study, the mortality index of patients developing complications while in the hospital was similar to that found in our study at 0.41. The use of the MSI-model confirms that faced with conditions of similar severity, a patient in Zanzibar has a 6-times higher chance of dying than the same patient in moderate mortality countries. In other words, the low quality of care given is responsible for much of the mortality.

In addition, the maternal near-miss approach provides a set of cases which through near-miss audits can be used to strengthen in-depth local analysis that is already performed in maternal death reviews. This improved analysis, however, will only be of practical use and have a positive impact on the quality of care if and when policy makers will act and assist in shaping conducive working conditions, mainly providing more qualified health workers [24].

The main limitation of this study is missing data, which is partly due to its retrospective character, because of data collection after patient discharge. Next to that, health care practice in Zanzibar does not yet guarantee thorough and complete diagnosing, monitoring and documentation, which, despite productive collaboration of the study’s investigators with the facility’s clinicians, leads to incomplete department data and patient files. Furthermore, we believe this to have led to underreporting of organ dysfunction markers, mainly of laboratory criteria but probably also of clinical and management criteria. We believe a prospective set-up of a future study is to be recommended, as that would likely lead to more complete data and a higher report rate of organ dysfunction markers, due to increased selection awareness. A last limitation of the study is the absence of maternal near-miss audits. Those would not only have given us a more complete representation of the clinical cases, but also would have stimulated reflection by health care workers themselves.

## Conclusion

The spectrum of severe maternal morbidity has been analysed in Zanzibar’s referral hospital using the WHO near-miss approach. Similar to other low-income, high-mortality settings, we find that quality of in-hospital care is low and failing to make a significant impact on progression of disease. Further in-depth analysis of near-miss cases needs to be performed, complementing maternal death reviews, to identify possible local solutions.

## Supporting information

S1 Table Markers for organ dysfunction, diagnosing maternal near-miss. (TIF)

S2 Table Comparison of maternal near-miss rate as a function of severity of the study population. (TIF)

S1 Database The data set used to reach the conclusions drawn in the manuscript. (XLSX)
