# Supplementary figures and images for: Severe maternal morbidity in Zanzibar’s referral hospital: Measuring the impact of in-hospital care
Source: PLoS One. 2017 Aug 23;12(8):e0181470. doi: 10.1371/journal.pone.0181470 (PMC5568340; doi:10.1371/journal.pone.0181470)

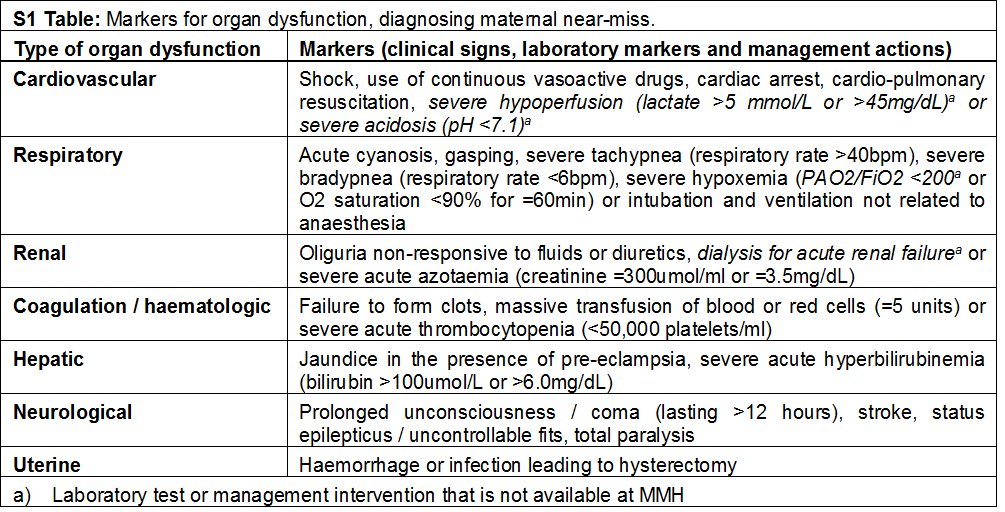

Supplement: S1 Table — (TIF) [file pone.0181470.s001.tif]

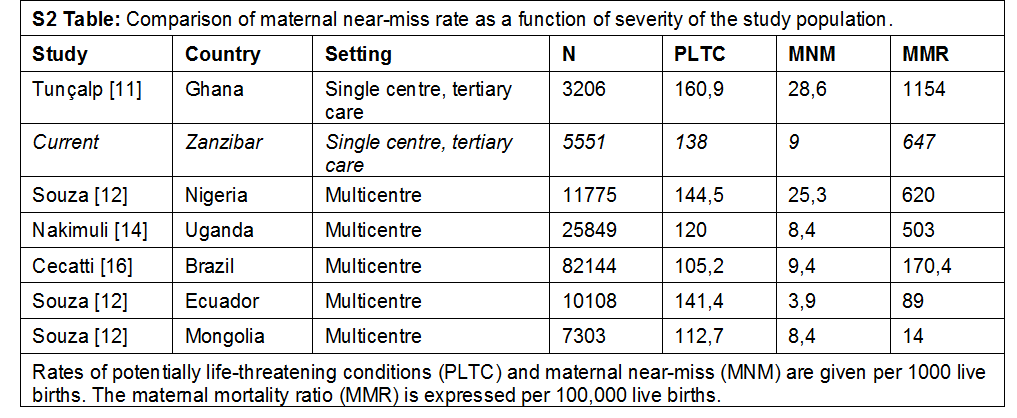

Supplement: S2 Table — (TIF) [file pone.0181470.s002.tif]
